# Radiofrequency ablation via catheter and transpapillary access in patients with cholangiocarcinoma (ACTICCA-2 trial) – a multicenter, randomized, controlled, open-label investigator-initiated trial

**DOI:** 10.1186/s12885-024-12693-w

**Published:** 2024-08-01

**Authors:** Constantin Schmidt, Antonia Zapf, Ann-Kathrin Ozga, Ali Canbay, Ulrike Denzer, Enrico N. De Toni, Ansgar W. Lohse, Kornelius Schulze, Thomas Rösch, Alexander Stein, Henning Wege, Johann von Felden

**Affiliations:** 1 I. Department of Medicine, University Medical Center Hamburg-Eppendorf, Martinistr. 52, 20246 Hamburg, Germany; 2 Institute of Medical Biometry and Epidemiology, University Medical Center Hamburg- Eppendorf, Hamburg, Germany; 3 Department of Internal Medicine, University Medical Center Knappschaftskrankenhaus Bochum, Bochum, Germany; 4 Department of Gastroenterology and Endocrinology, University Medical Center Marburg, Marburg, Germany; 5 Department of Medicine II and Comprehensive Cancer Center Munich, University Medical Center Ludwig-Maximilian-University Munich, Munich, Germany; 6 Department of Interdisciplinary Endoscopy, University Medical Center Hamburg-Eppendorf, Hamburg, Germany; 7 University Cancer Center Hamburg, University Medical Center Hamburg-Eppendorf, Hamburg, Germany

**Keywords:** Cholangiocarcinoma, CCA, Biliary tract cancer, Klatskin tumor, Biliary ablation, Bile duct stenting, Radiofrequency ablation, Palliative chemotherapy, Immunotherapy

## Abstract

**Background:**

Despite the recent advances in cancer treatment, the therapeutic options for patients with biliary tract cancer are still very limited and the prognosis very poor. More than 50% of newly diagnosed patients with biliary tract cancer are not amenable to curative surgical treatment and thus treated with palliative systemic treatment. Malignant bile duct obstructions in patients with perihilar and/or ductal cholangiocarcinoma (CCA) represents one of the most important challenges in the management of these patients, owning to the risk represented by developing life-threatening cholangitis which, in turn, limits the use of systemic treatment. For this reason, endoscopic stenting and/or bile duct decompression is the mainstay of treatment of these patients. Data on efficacy and safety of adding radiofrequency ablation (RFA) to biliary stenting is not conclusive. The aim of this multicenter, randomized trial is to evaluate the effect of intraductal RFA prior to bile duct stenting in patients with unresectable perihilar or ductal CCA undergoing palliative systemic therapy.

**Methods/Design:**

ACTICCA-2 is a multicenter, randomized, controlled, open-label, investigator-initiated trial. 120 patients with perihilar or ductal CCA with indication for biliary stenting and systemic therapy will be randomized 1:1 to receive either RFA plus bile duct stenting (interventional arm) or bile duct stenting alone (control arm). Patients will be stratified by trial site and tumor location (perihilar vs. ductal). Both arms receive palliative systemic treatment according to the local standard of care determined by a multidisciplinary tumorboard. The primary endpoint is time to first biliary event, which is determined by an increase of bilirubin to > 5 mg/dl and/or the occurrence of cholangitis leading to premature stent replacement and/or disruption of chemotherapy. Secondary endpoints include overall survival, safety according to NCI CTCAE v5, quality of life assessed by questionnaires (EORTC QLQ-C30 and QLQ-BIL21), clinical event rate at 6 months after RFA and total days of over-night stays in hospital. Follow-up for the primary endpoint will be 6 months, while survival assessment will be continued until end of study (maximum follow-up 30 month). All patients who are randomized and who underwent endoscopic stenting will be used for the primary endpoint analysis which will be conducted using a cause-specific Cox proportional hazards model with a frailty for trial site and fixed effects for the treatment group, tumor location, and stent material.

**Discussion:**

ACTICCA-2 is a multicenter, randomized, controlled trial to assess efficacy and safety of adding biliary RFA to bile duct stenting in patients with CCA receiving palliative systemic treatment.

**Trial registration:**

The study is registered with ClinicalTrials.gov (NCT06175845) and approved by the local ethics committee in Hamburg, Germany (2024-101232-BO-ff). This manuscript reflects protocol version 1 as of January 9th, 2024.

**Graphical Abstract:**

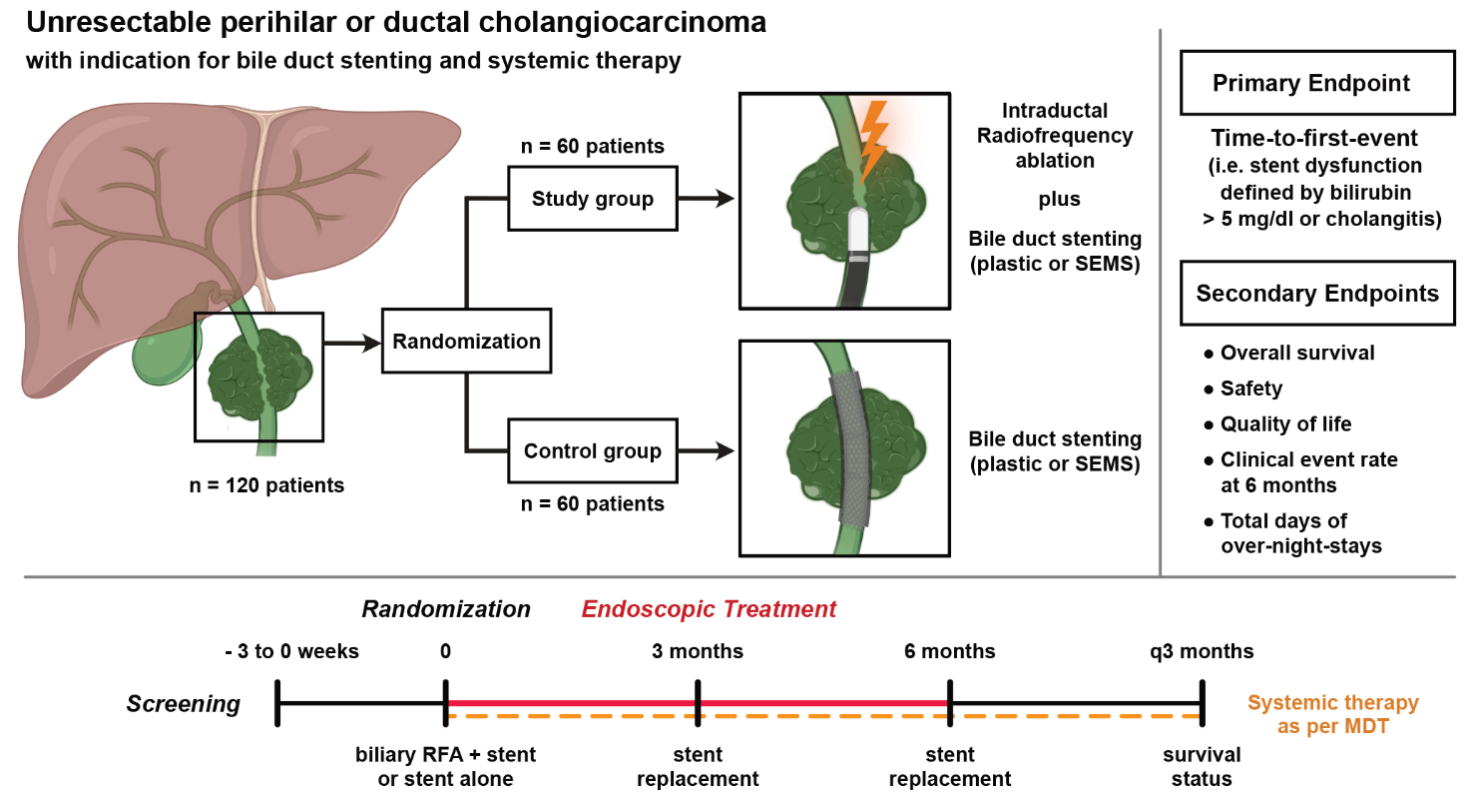

**Supplementary Information:**

The online version contains supplementary material available at 10.1186/s12885-024-12693-w.

## Background

### Epidemiology of biliary tract cancer and medical problem

Despite new therapeutic options to treat malignancies, mortality for liver cancer, and particularly for biliary tract cancer, is rising and the prognosis is very poor [1]. At the same time, therapeutic options remain very limited, particularly for patients with biliary tract cancer not amenable to curative surgical resection. [1, 2]. The age-standardized incidence rate of cholangiocarcinoma (CCA) in western countries is 0.3–3.5 cases per 100 000. However, in Asian countries the incidence is up to 40 times higher, reaching 85 cases per 100 000 in Thailand. Especially the incidence of intrahepatic CCA has been steadily increasing in most Western countries, and less than 50% of these newly diagnosed patients are amenable to curative surgical therapy [2].

Regarding patients with unresectable CCA, systemic therapy unequivocally represents the standard of care [2]. However, patients with perihilar and/or ductal CCA are in danger of developing life-threatening cholangitis due to malignant bile duct obstruction and often require prophylactic stenting and/or bile duct decompression before and during the administration of palliative chemotherapy [1, 2]. In a large proportion of these patients (approximately 30% in 3 months), stent dysfunction and episodes of cholangitis occur resulting in a delay of systemic therapy. Therefore, additional intraductal therapies, specifically photodynamic therapy (PDT) [3] and endobiliary radiofrequency ablation (RFA) [4], have been investigated for the management of malignant biliary obstructions and to improve stent patency. Despite a convincing mechanistic rationale for these intraductal interventions and a huge medical need, evidence comparing RFA plus stenting and stenting alone is scarce and/or conflicting, particularly regarding effects in the setting of palliative chemotherapy [5–9].

### Treatment modalities for unresectable biliary tract cancer

Based on an investigator-initiated trial (IIT) by Valle et al., including more than 750 patients, palliative chemotherapy with gemcitabine and cisplatin has been the standard of care (SOC) for patients with unresectable biliary tract cancer [10–12].

Recently, the phase III clinical trial TOPAZ-1 evaluating the PD-L1 antibody durvalumab in combination with gemcitabine and cisplatin against gemcitabine and cisplatin alone in patients with unresectable biliary tract cancer has met its primary endpoint with increased overall survival (OS) for the triple combination (12.8 vs. 11.5 months, hazard ratio of 0.8 (95% confidence interval [CI]: 0.66, 0.97) [13]. In contrast, data regarding the efficacy and safety of adding PDT or RFA to biliary stenting in patients with biliary obstruction who are in need for systemic therapy, is limited and conflicting.

A recent meta-analysis from 2021 assessed the role of PDT, RFA, and biliary stenting alone in the palliative treatment of unresectable extrahepatic CCA [14]. A total of 1149 patients underwent treatment with PDT (33 studies), 545 with RFA (22 studies), and 452 patients with stent-only strategy. The pooled median survival with PDT, RFA, and stent-only groups was 11.9 (95% CI: 10.7–13.1) months, 8.1 (95% CI: 6.4–9.9) months, and 6.7 (95% CI: 4.9–8.4) months, respectively. However, the subgroup of endoscopic RFA (235 patients, excluding percutaneous ablation) yielded a median OS of 12 (95% CI: 9.8–14.3) months, similar to PDT. The study reported a pooled time of stent patency for PDT, RFA, and stent-only groups of 6.1 (95% CI: 4.2–8) months, 5.5 (95% CI: 4.2–6.7) months, and 4.7 (95% CI: 2.6–6.7) months, respectively. Regarding adverse events, the pooled rates of cholangitis and liver abscess were highest among patients undergoing PDT compared to RFA and stent-only groups (cholangitis; 23.4%, 9.5%, 15.5%; liver abscess: 4.9%, 2.6%, 2.1%), whereas the pooled rates of hemobilia were comparable between PDT, RFA, and stent-only (5.4%, 4.3%, 3.2%). However, it is important to emphasize that large randomized studies directly comparing these procedures are lacking [15].

Early studies investigating the effects of RFA reported relatively high rates of complications and adverse events, which was likely due to the relatively new procedure. In a retrospective study from 2014, 19 endoscopic ablations were performed in 12 patients with unresectable malignant hilar bile duct obstruction [16]. Four of the 12 patients also received chemotherapy. The median OS from the time of the first RFA was 6.4 months. In this cohort, biliary bleeding was observed in 3 cases and severe cholangitis in another 3 patients. Along this line, a collaborative registry trial from the U.S. also reported a high rate of adverse events (10%) in 69 patients, of whom 78% also received chemotherapy, again with mixed aetiologies of malignant biliary obstructions undergoing intraductal RFA [17]. A small study in the U.S. compared OS rates in patients receiving RFA (*n* = 16) or PDT (*n* = 32) and did not report a statistically significant difference (9.6 months for RFA versus 7.5 months for PDT, *p* = 0.799) for these interventions [18]. In this study however, patients who underwent RFA (compared to PDT) had a lower mean number of plastic stents placed per month (0.45 vs. 1.10, *p* = 0.001), but had more episodes of stent occlusion (0.06 vs. 0.02, *p* = 0.008) and cholangitis (0.13 vs. 0.05, *p* = 0.008) per month. In addition, the complication rate in this study (stent occlusion and/or cholangitis requiring premature stent replacement) interfering with the administration of palliative chemotherapy was 7–19% per month or 20–40% in 3 months in patients receiving additional transductal interventions (RFA or PDT). Finally, a recent study compared adverse events and short-term effects of RFA (*n* = 14) vs. PDT (*n* = 20) in perihilar CCA [19]. In the PDT group, a significantly higher number of premature stent replacements (< 3 months) after the first intervention was noticed in comparison with the RFA group (65% vs. 29%, *p* < 0.01). Between the first and fifth stent replacement, post-interventional adverse events tended to occur more frequently in patients with PDT than with RFA (40% vs. 21%, *p* = 0.277).

More recently, three randomized controlled trials were reported. Most recently, a multicenter study including patients with CCA and pancreatic cancer with malignant biliary obstruction by Jarosova et al. (2023) compared endoluminal RFA plus stenting and stenting alone. In this study, OS did not differ significantly between the RFA group and the control group among patients with CCA (10.5 vs. 10.6 months, *n* = 85, *p* = 0.58), and no benefit was seen in the RFA group with regards to stent patency (40% vs. 36% at 12 months), quality of life, and adverse event rate [6]. The study was therefore prematurely terminated due to futility. Noteworthy, the RFA group contained more patients with distal CCA (18% vs. 10%), which is considered to have a worse prognosis compared to hilar CCA, and less patients with concomitant (9% vs. 20%) or adjuvant (20% vs. 37%) anti-cancer therapy rendering a potential bias in the survival analysis. In contrast, studies by Gao et al. and Yang et al. reported improved OS when comparing RFA plus stenting vs. stenting alone. Gao et al. performed a multicenter randomized trial, including 47 patients with hilar CCA and 100 patients with distal CCA, and reported an OS of 13.3 months in the RFA group vs. 9.2 months in the control group (hazard ratio 0.546, *p* < 0.001). However, no significant differences were found regarding jaundice control or stent patency duration between the groups [7]. Noteworthy, this study was limited to patients who were not eligible for systemic therapy, thus reflecting a different patient population compared to our study design. In line with this, Yang et al. performed a single-center randomized trial in patients with perihilar or distal CCA (*n* = 65) and reported an OS of 13.2 vs. 8.3 months (*p* < 0.001) for RFA plus stenting vs. stenting alone [9]. However, the latter trial did not include data on systemic chemotherapy as SOC in these patients. In conclusion, evidence is scarce and inconsistent and thus comparing RFA plus stenting vs. stenting alone in the context of systemic chemotherapy in patients with unresectable CCA is strongly required.

### Study rationale

As discussed before, conflicting data has been reported for patients receiving intraductal therapy (RFA or PDT) for malignant bile duct obstructions compared to stenting alone. RFA seems to be equally effective regarding OS and stent patency compared to PDT, but with a lower number of complications [14, 20]. Taken together, it is reasonable to assume that the improved OS in patients treated with endoscopic RFA compared to stent alone is likely due to the longer stent patency and fewer stent replacements which prevent life-threatening biliary complications and interruptions of systemic chemotherapy. However, these findings are mainly derived from retrospective studies and early reports on complications (hemobilia, cholangitis, stent obstruction) make it difficult to judge, whether intraductal RFA in addition to stenting of malignant biliary obstructions is beneficial in patients with unresectable perihilar and/or ductal CCA, particularly in the setting of palliative chemotherapy as SOC [5, 15]. Unfortunately, standard systemic therapy was not included in any of the recent randomized trials [6, 9, 21].

Data on prognostic factors for CCA are rare. Within the current trial, tumor tissue (if available), bile fluid, plasma, and buffy coat will be collected with the clinical data. An allocation database will be established gathering the data of the available patient samples at each study site to enable translational research.

### Study objective

The primary objective of this study is to evaluate the efficacy of intraductal RFA prior to stent placement compared to stent placement alone in patients with perihilar or ductal CCA who receive systemic treatment. The secondary objectives are OS, safety, quality of life (QoL), clinical event rate, and days of overnight hospital stays.

### Methods/Design

The ACTICCA-2 study is a multicenter, prospective, randomized, open-label trial designed to assess the clinical performance of intraductal RFA plus biliary stenting in comparison to stenting alone both in combination with palliative systemic treatment in patients with CCA. A total of 120 patients will be assigned to the trial and randomized 1:1 to receive either RFA plus biliary stenting or stenting alone (*n* = 60 per arm). Patients will be followed up for a minimum of 6 months. The total study duration will be 30 months. Patient recruitment will be initiated in August 2024.

### Patient selection and randomization

Patients will be enrolled into the trial according to the eligibility criteria (Table 1). Inclusion and exclusion criteria are based on the main selection criteria for patients with unresectable perihilar and/or ductal CCA who are recommended to receive bile duct stenting and palliative systemic therapy by current guidelines [2]. In detail, these criteria reflect the patient’s clinical status including potential safety concerns regarding the endoscopic intervention and administration of systemic chemotherapy. Performance status 0–1 according to the Eastern Cooperative Oncology Group (ECOG) is a well-established cut-off for the eligibility of patients to receive systemic therapy and invasive procedures, such as endoscopic retrograde cholangiopancreatography (ERCP). Patients will be excluded if prior RFA and/or multiple stenting for CCA has been performed during the past 3 months to rule out competing effects on the outcome analysis (first stent replacement or placement of the first stent for tumor-related bile duct obstruction within 3 months prior to study inclusion is allowed). Patients with concomitant diseases or malignancies with limited prognosis or high likelihood of study discontinuation (e.g. severe or uncontrolled cardiovascular disease, such as congestive heart failure New York Heart Association (NYHA) III or IV, unstable angina pectoris, myocardial infarction within ≤ 3 months, and/or significant arrhythmias) will be excluded. This is in line with clinical eligibility criteria for the administration of bile duct stenting and subsequent administration of systemic therapy. Taken together and considering a relatively large sample size and a multicenter study design, the study population is representative, and results are generalizable to the target population of interest.

**Table 1:** Eligibility criteria for the ACTICCA-2 enrollment phase

Unresectable perihilar and/or ductal CCA with indication for bile duct stenting
Signed written informed consent
ECOG performance status 0–1
Age ≥ 18 years
Eligibility for palliative systemic therapy based on clinical and laboratory parameters (except hyperbilirubinemia) as determined by the local MDT
No prior RFA for CCA
No repeated bile duct stenting for > 3 months (trial inclusion is possible upon first stent replacement or initial stent placement)
No concomitant disease or malignancy interfering with the study procedure or efficacy outcome measures, particularly no severe or uncontrolled cardiovascular disease (congestive heart failure NYHA III or IV, unstable angina pectoris, myocardial infarction within ≤ 3 months, significant arrhythmias) and no psychiatric disorders precluding understanding of information of trial related topics and giving informed consent

*CCA*: cholangiocarcinoma, *ECOG*: Eastern Cooperative Oncology Group, *RFA*: radio frequency ablation, *NYHA*: New York Heart Association Functional Classification, *MDT*: multidisciplinary team

Randomization to study treatment should occur within three weeks after eligibility criteria have been met. Study subjects will be randomized by the electronic CRF to arm A (intervention group) or B (control group) in 1:1 ratio, stratified by trial site and tumor location (perihilar vs. ductal). The procedure itself (i.e., endoscopic stenting with or without RFA) and randomization are on the same day.

### Treatment and trial procedures

**Fig. 1:**
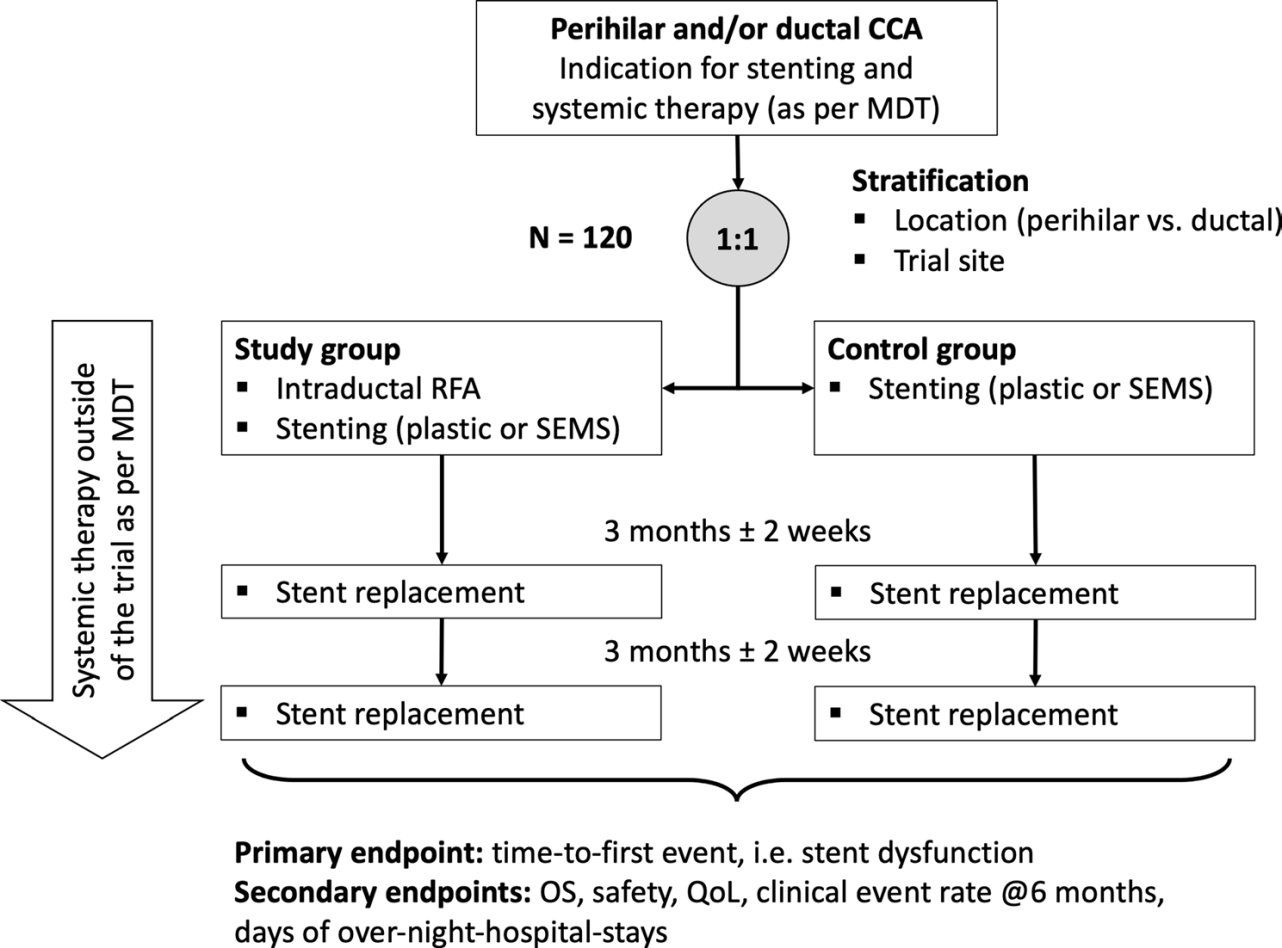
ACTICCA-2 trial design. *CCA*: cholangiocarcinoma, *MDT*: multidisciplinary team, *RFA*: radiofrequency ablation, *SEMS*: self-expanding metal stent, *OS*: overall survival, *QoL*: quality of life

Systemic chemotherapy will be administered outside of the trial as per local standard (Fig. 1).

As per study protocol, the selection of the RFA device is up to the discretion of the investigator, given approval in this context. The device provides bipolar energy to perform partial or complete ablation of tissue in the biliary tract. The RFA procedure will be performed only once at the beginning of the study. The extent of RFA for each individual patient will be decided by the investigator during the procedure. Of importance, trial site is one of the stratification criteria for randomization of patients to account for a possible procedure-related effects. Subsequent stent replacements will be carried out without additional RFA regardless of patient randomization to the intervention or control group.

All patients in the trial will receive endoscopic stenting and palliative chemotherapy as SOC as is indicated by local standards and as recommended in current guidelines [2]. Patients receiving liver-directed local therapy for hilar lesions, such as internal or external radiation therapy or chemosaturation will be censored at time of treatment if already enrolled in the trial. Radiation therapy to distant tumor sites (e.g. bone) is allowed. There are no restrictions on medications or other treatments, including full access to palliative care and symptom management (including pain control) throughout the trial.

### Arm A (intraductal RFA plus biliary stenting)

Patients assigned to the study arm will receive systemic treatment and bile duct stenting with plastic or metallic stents with intraductal RFA of malignant bile duct obstructions employing a CE-certified RFA-catheter prior to stent placement. RFA will be applied once.

### Arm B (control: biliary stenting alone)

Patients assigned to the control arm will receive systemic treatment and bile duct stenting with plastic or metallic stents alone.

### Assessments for screening, treatment and follow-up

An overview of trial assessment is displayed in Table 2.

**Table 2:** Overall study schedule overview

		STUDY PERIOD				
		Screening/ Enrollment	Randomization, Allocation + Visit 1	Post-allocation		
				Visit 2	Visit 3	Follow-up
Timepoint		-3–0 weeks	0	3 months (± 2 weeks)	6 months (± 2 weeks)	q3 months (± 2 weeks)
ENROLMENT:	Eligibility screen	X				
	Informed consent	X				
	Medical history (a)	X				
	Lab tests (b)	X	X	X	X	
INTERVENTIONS:	ERCP + stent		X	X (c)	X (c)	
	ERCP + stent + RFA (d)		X			
ASSESSMENTS:	Assessment primary endpoint (e)			X	X	
	Safety (f)		X	X	X	
	QoL (g)		X	X	X	
	Clinical event rate @ 6 months (h)				X	
	Cumulative days of overnight-hospital stays				X	
	Systemic therapy (i)		X	X	X	
	Survival status			X	X	X (k)

^a)^ Demographic data, baseline tumor data, concomitant diseases, current medication, prior endoscopic procedures

^b)^ CBC, INR, GGT, AP, AST, ALT, bilirubin total and direct, creatinine, Sodium, Potassium, CRP

^c)^ Only in patients who did not experience primary endpoint until then

^d)^ Only in patients randomized in the study group

^e)^ Time-to-first event (for 6 months follow up), i.e. stent dysfunction defined by bilirubin > 5 mg/dl and/or cholangitis

^f)^ Terminology and grading according to NCI CTCAE v5

^g)^ EORTC QLQ-C30 and EORTC QLQ-BIL21

^h)^ Cumulative rate of clinical events @ 6 months, such as stent dysfunction defined by bilirubin > 5 mg/dl and/or cholangitis

^i)^ Data collection regarding administration and complications of systemic therapy (administered as SOC)

^k)^ Per telephone contact

#### Screening

Within 3 weeks prior to randomization/start of first treatment (i.e. stenting with or without RFA) the following will be assessed/obtained:

- Signed written informed consent.
- Medical history including previous cancer history, cancer treatment, concomitant diseases, current medication, prior endoscopic procedures.
- Demographics.
- ECOG performance status.
- Laboratory tests: Hematology panel (hemoglobin, platelets, WBC and WBC differential with neutrophils, lymphocytes, monocytes) chemistry panel (sodium, potassium, calcium, creatinine, total and direct bilirubin, alkaline phosphatase, 𝛾GT, ALT, AST, CrP, INR, aPTT, PT) and CA 19 − 9, CEA (optional).

#### Randomization and treatment (visit 1)

After enrolment, patients will be randomized (1:1) to either receive intraductal RFA followed by bile duct stenting or bile duct stenting alone. Additionally, the following will be assessed for baseline:

- Laboratory tests as shown above (only if screening lab values are > 7 days old).
- Safety (terminology and grading according to NCI CTCAE v5 [see suppl. material])
- Data collection regarding administration and complications of systemic therapy (administered as SOC).
- Quality of life (QoL) assessment using the European Organisation for Research and Treatment of Cancer (EORTC) questionnaire QLQ-C30 [see suppl. material Fig. 1] and the module BIL21 [see suppl. material Fig. 2].

#### Visit 2 & 3

The following will be assessed at three and six months (each +/- 2 weeks) after randomization:

- Laboratory tests as shown above.
- ERCP and stent replacement (within regular clinical management of the patients), not mandatory for patients who have experienced the primary endpoint of stent dysfunction, resulting in a change of timeline regarding stent replacement.
- Safety, data collection regarding administration and complications of systemic therapy (SOC), QoL, days of overnight hospital stays, and survival status.

#### Survival follow-up

Following Visit 3, every three months (+/- 2 weeks), the survival status will be assessed remotely, e.g., via telephone contact, until the end of the study.

### Endpoint definition

The primary endpoint is time to first event, i.e. stent dysfunction defined by bilirubin > 5 mg/dl and/or cholangitis (fever > 38.5 °C and/or increase in C-reactive protein (CrP) by at least 3-fold upper limit of normal and at least 20% from baseline without extrahepatic focus and absence of tumor progression) leading to premature stent replacement and/or disruption of chemotherapy. This endpoint is clinically relevant and paramount for the management of patients with perihilar and/or ductal CCA and represents a generally agreed core outcome for this population. Because systemic treatment discontinuation due to biliary complications is so critical for these patients, these endpoints are proposed by current guidelines as surrogates for survival [2]. According to recently published studies on this topic, the event rate at 6 months in the control group is estimated to be 50% [14, 20]. Our study aims to detect a clinically relevant reduction of the rate by 50% in the RFA group to 25%, resulting in a number needed to treat (NNT) of 4. This event reduction has to be achieved in the setting of palliative systemic therapy and seems reasonable considering the previously reported beneficiary effects of RFA on stent patency and OS [7, 9, 14, 20]. Importantly, all possible events will be reviewed by a blinded and independent endpoint-review committee (ERC), leading to a prospective randomized open blinded endpoint (PROBE) design.

The secondary endpoints will include OS, QoL, clinical event rate at 6 months, total days of over-night-hospital-stays during the trial period as well as safety (terminology and grading according to NCI CTCAE v5). Median OS will be calculated as a secondary outcome to generate data for larger clinical trials targeting OS as a primary endpoint. Following the fixed study follow-up period of 6 months, patients or primary care providers will be contacted every 3 months by telephone call. QoL will be assessed with EORTC QLQ-C30 [suppl. material Fig. 1] and EORTC QLQ-BIL21 [suppl. material Fig. 2] questionnaires at each of the three on-site study visits. Clinical events as defined by the primary endpoint, i.e. stent dysfunction leading to premature stent replacement and/or disruption of chemotherapy, will be counted at 6 months follow-up and reported as a time-independent clinical event rate. In addition, cumulative over-night-hospital-stays within the fixed 6 months follow-up period will be counted. Moreover, assessing the safety of intraductal RFA is a major aim of the study and will be investigated as a secondary endpoint. RFA-related events will be examined, documented, and graded according to NCI CTCAE v5.

### Quality assurance and safety

Patient data will be collected in electronic case report forms (CRF). Three independent experts will be appointed to serve as an Independent Data Monitoring Committee (IDMC) and give expert advice and supervision entirely independent of the applicants and the medical institutions involved. The IDMC will conduct thorough safety assessments throughout the study and will review all RFA-related events and decide on trial continuation after every 10th RFA procedure. A “harm rate” of ≥ 20% (≥ 6 RFA-related grade ≥ 4 events among the first 30 patients in the intervention group) will be suggested to the IDMC as critical.

The ERC will be responsible to blindly review and to verify countable events in both study arms to reduce possible biases caused by lack of blinding of the investigators to the RFA procedure. In addition, regular monitoring visits will be conducted by the AIO-Studien-gGmbH to ensure protocol adherence of the sites. A safety event (adverse event (AE) as per ICH-good clinical practice (GCP)) is defined as any untoward medical occurrence or experience in a subject or clinical investigation subject. Worsening of a pre-existing medical condition (e.g. diabetes, migraine headaches, gout) will be considered a safety event if there is either an increase in severity, frequency, or an association with significantly worse outcomes. A possible safety event will be graded according to CTCAE v5 and will be reviewed by the investigator and is properly captured in the subjects’ medical records and the electronic CRF. CTCAE grade 1 and 2 will only be regarded a safety event, if judged as “clinically relevant” by the investigator or if interfering with treatment administration. CTCAE grade 3 and 4 will always be regarded as safety events.

### Sample size calculation and statistical analysis

The sample size of 120, i.e., 60 patients per group, was calculated based on the two-sided log-rank test for time to first event analysis. Thereby, a power of 80% can be reached under the following assumptions and with a two-sided significance level of 5%. Based on previous reports [7, 9, 14, 20], the event rate at 6 months in the control group was estimated to be 50% and 25% in the intervention group resulting in a hazard ratio (HR) of 0.415. The follow-up will be fixed at 6 months. Furthermore, it was assumed that there will be a low loss to follow-up of 2.5% over six months and all patients stay within their treatment group within the observational period. PASS version 16.0.3 was used for the calculation of the sample size.

Descriptive analysis for baseline characteristics will be performed giving mean, standard deviation, median, and inter-quantile range for continuous data as well as counts and percentages for categorical data. Tests of statistical significance will not be undertaken for baseline characteristics; rather the clinical importance of any imbalance will be noted [22]. A modified ITT (mITT) population will be used for the primary endpoint analysis. The mITT population consists of all patients who will be randomized in the ACTICCA-2 trial and who will undergo endoscopic stenting.

Primary endpoint analysis will be conducted using a cause-specific Cox proportional hazards model comparing the two treatment groups regarding time to first event within 6 months with trial site as random effect (frailty) and tumor location (perihilar vs. ductal) and stent material (plastic vs. metallic) as fixed effects. An adjusted HR for the treatment effect with corresponding 95% CI and p-value will be given as well as cumulative incidences with 95% CIs. The secondary endpoint OS will be analyzed with the Cox proportional hazards model with a frailty for trial site and adjusting for stratification variable tumor location and the variable stent material. Mixed models will be used for further secondary endpoints (linear, logistic, or negative binomial as appropriate).

The mITT population will also be used for the secondary endpoint analysis. As a sensitivity analysis for the primary event of interest, results of the model by Fine and Gray will be given to take the competing event “death” into account. Cause-specific time-to-event analysis will be conducted for the components of the primary endpoint. Explorative subgroup analyses will be conducted in the same manner as for the primary outcome. Further sensitivity analysis will be conducted for the primary outcome, i.e., analysis for the per protocol population. The per protocol population includes all patients without major protocol violations. Furthermore, safety analyses will be conducted by the ERC/IDMC throughout the trial to decide about trial continuation after every 10th RFA procedure. Interim analyses are not planned. Missing values will not be imputed. Only the primary endpoint is analyzed in confirmatory manner with a two-sided significance level of 5% and all other analysis are considered explorative. Hence, no adjustment for multiple testing is conducted. A detailed statistical analyses plan will be prepared and finalized before the randomization code is broken. Data will be analyzed according to the Consolidated Standards of Reporting Trials (CONSORT) statement [23]. Statistical analyses will be carried out with an established statistical software such as SAS (Cary: SAS Institute Inc.; 2013.), Stata (College Station: StataCorp LLC.; 2021), R (Vienna: R Foundation for Statistical Computing; 2023.), or SPSS (Armonk: IBM Corp; 2021.).

### Translational research

Within the current trial, tumor tissue, if available, bile fluid, plasma, and buffy coat will be collected to establish a biobank. Participation is optional. The aim of this translational research project is to assess the prognostic and predictive impact of different biomarkers in cholangiocarcinoma.

### Electronic supplementary material

Below is the link to the electronic supplementary material.

Supplementary Material 1

## Data Availability

Not applicable as this manuscript does not contain any original data. Data generated throughout this study will be published separately.

## Abbreviations

AE: Adverse event
BIL21: Module Questionnaire for cholangiocarcinoma and gallbladder cancer
CCA: Cholangiocarcinoma
CrP C-: reactive protein
CI: Confidence interval
CRF: Case Report Form
CONSORT: Consolidated Standards of Reporting Trials
CTCAE: Terminology Criteria for Adverse Events
ECOG: Eastern Cooperative Oncology Group
EORTC QLQ-C30: European Organization for Research and Treatment of Cancer Quality of Life Questionnaire-Core 30
ERC: Endpoint Review Committee
ERCP: Endoscopic Retrograde Cholangiopancreaticography
GCP: Good Clinical Practice
HR: Hazard ratio
ICF: Informed consent form
IDMC: Independent Data Monitoring Committee
IEC: Independent Ethics Committee
IIT: Investigator-initiated trial
ITT: Intention-to-treat
mITT: modified intention-to-treat
MDT: Multidisciplinary Team
NCI: National Cancer Institute
NNT: Number-needed-to-treat
NYHA: New York Heart Association
OS: Overall survival
PDT: Photodynamic therapy
QoL: Quality of life
RDE: Remote data entry
RFA: Radiofrequency ablation
SAE: Serious adverse event
SDV: Source Data Verification
SOC: Standard of care
PROBE: Prospective randomized open blinded evaluation
TNM: Classification of malignant tumors
