# Supplementary material for: Radiofrequency ablation via catheter and transpapillary access in patients with cholangiocarcinoma (ACTICCA-2 trial) – a multicenter, randomized, controlled, open-label investigator-initiated trial
Source: BMC Cancer. 2024 Aug 1;24:931. doi: 10.1186/s12885-024-12693-w (PMC11293169; doi:10.1186/s12885-024-12693-w)
Supplement: Supplementary file 1 — Supplementary Material 1 [file 12885_2024_12693_MOESM1_ESM.docx]

# Supplementary material

## Common Terminology Criteria for Adverse Events (CTCAE)

In the present study, adverse events and/or adverse drug reactions will be recorded according to the Common Terminology Criteria for Adverse Events (CTCAE), version 5.

At the time this protocol was issued, the full CTC document was available on the NCI web site, at the following address:

<https://ctep.cancer.gov/protocoldevelopment/electronic_applications/docs/CTCAE_v5_Quick_Reference_5x7.pdf>

Another option is via the EORTC Headquarters web site [www.eortc.be](http://www.eortc.be), which provides a link to the appropriate CTC web site.

## Suppl. material figure 1 – EORTC QLQ-C30

## Suppl. material figure 2 – EORTC QLQ-BIL21
